# A Live Attenuated Influenza Vaccine Elicits Enhanced Heterologous Protection When the Internal Genes of the Vaccine Are Matched to Those of the Challenge Virus

**DOI:** 10.1128/JVI.01065-19

**Published:** 2020-01-31

**Authors:** Andrew Smith, Laura Rodriguez, Maya El Ghouayel, Aitor Nogales, Jeffrey M. Chamberlain, Katherine Sortino, Emma Reilly, Changyong Feng, David J. Topham, Luis Martínez-Sobrido, Stephen Dewhurst

**Affiliations:** a Department of Microbiology and Immunology, University of Rochester, Rochester, New York, USA; b Medical Scientist Training Program, University of Rochester, Rochester, New York, USA; c David H. Smith Center for Vaccine Biology and Immunology, University of Rochester, Rochester, New York, USA; d Department of Biostatistics and Computational Biology, University of Rochester, Rochester, New York, USA; University of North Carolina at Chapel Hill

**Keywords:** influenza, live attenuated influenza vaccine, LAIV, inactivated influenza vaccine, IIV, master donor virus, MDV, heterologous immunity and protection

## Abstract

Influenza A virus (IAV) causes significant morbidity and mortality, despite the availability of viral vaccines. The efficacy of live attenuated influenza vaccines (LAIVs) has been especially poor in recent years. One potential reason is that the master donor virus (MDV), on which all LAIVs are based, contains either the internal genes of the 1960 A/Ann Arbor/6/60 or the 1957 A/Leningrad/17/57 H2N2 viruses (i.e., they diverge considerably from currently circulating strains). We previously showed that introduction of the temperature-sensitive (*ts*) residue signature of the AA/60 MDV into a 2009 pandemic A/California/04/09 H1N1 virus (Cal/09) results in only 10-fold *in vivo* attenuation in mice. We have previously shown that the *ts* residue signature of the Russian A/Leningrad/17/57 H2N2 LAIV (Len LAIV) more robustly attenuates the prototypical A/Puerto Rico/8/1934 (PR8) H1N1 virus. In this work, we therefore introduced the *ts* signature from Len LAIV into Cal/09. This new Cal/09 LAIV is *ts in vitro*, highly attenuated (*att*) in mice, and protects from a lethal homologous challenge. In addition, when our Cal/09 LAIV with PR8 hemagglutinin and neuraminidase was used to vaccinate mice, it provided enhanced protection against a wild-type Cal/09 challenge relative to a PR8 LAIV with the same attenuating mutations. These findings suggest it may be possible to improve the efficacy of LAIVs by better matching the sequence of the MDV to currently circulating strains.

**IMPORTANCE** Seasonal influenza infection remains a major cause of disease and death, underscoring the need for improved vaccines. Among current influenza vaccines, the live attenuated influenza vaccine (LAIV) is unique in its ability to elicit T-cell immunity to the conserved internal proteins of the virus. Despite this, LAIV has shown limited efficacy in recent years. One possible reason is that the conserved, internal genes of all current LAIVs derive from virus strains that were isolated between 1957 and 1960 and that, as a result, do not resemble currently circulating influenza viruses. We have therefore developed and tested a new LAIV, based on a currently circulating pandemic strain of influenza. Our results show that this new LAIV elicits improved protective immunity compared to a more conventional LAIV.

## Abstract (precis)

Seasonal influenza infection remains a major cause of disease and death, underscoring the need for improved vaccines. Among current influenza vaccines, the live attenuated influenza vaccine (LAIV) is unique in its ability to elicit T-cell immunity to the conserved internal proteins of the virus. Despite this, LAIV has shown limited efficacy in recent years. One possible reason is that the conserved, internal genes of all current LAIVs derive from virus strains that were isolated between 1957 and 1960 and that, as a result, do not resemble currently circulating influenza viruses. We have therefore developed and tested a new LAIV, based on a currently circulating pandemic strain of influenza. Our results show that this new LAIV elicits improved protective immunity compared to a more conventional LAIV.

## INTRODUCTION

**FIG 1:**
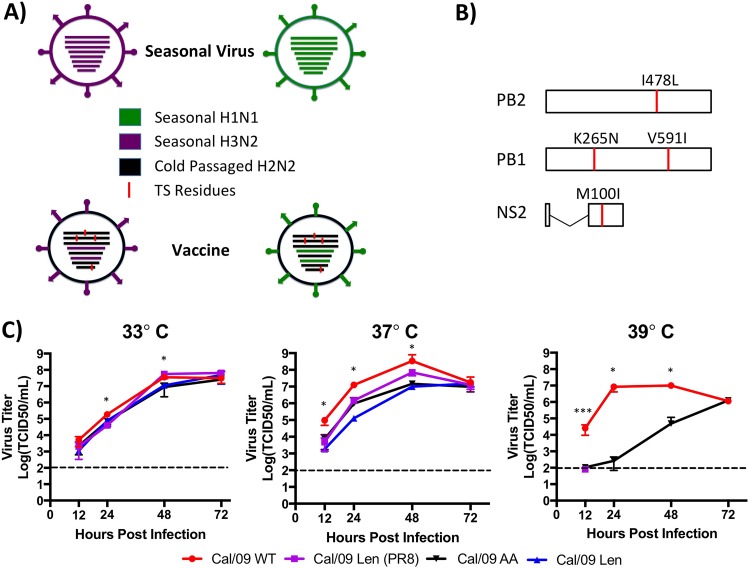
Cal/09 Len has a more robust *ts* phenotype than Cal/09 AA. (A) Schematic representation of currently licensed Russian LAIV. Gene segments from a seasonally circulating strain (green), as well as gene segments from cold-passaged H2N2 virus (black), and *ts* mutations (red) are indicated in the center of virions arranged in order of segment length (PB2, PB1, PA, HA, NP, NA, M, and NS from longest to shortest). Surface antigens from seasonal strains are also denoted on the periphery of virions (green). (B) Schematic representation of viral genes PB2, PB1, and NS. Polymerase subunits PB2 (top) and PB1 (middle), as well as the nuclear export protein NS2 (bottom) of Cal/09 containing the mutations responsible for the temperature-sensitive (*ts*) phenotype of the Leningrad-based LAIV (red), are indicated. (C) Viral growth kinetics. MDCK cells were infected at a low MOI of 0.001 with recombinant Cal/09 WT (red), Cal/09 Len (blue), Cal/09 Len (PR8) (purple), and Cal/09 AA (black) viruses and then incubated at 33°C (left), 37°C (middle), or 39°C (right). Tissue culture supernatants from infected cells were collected at the indicated times postinfection, and viral titers were determined as the TCID_50_/ml. Data represent the means ± the SD of results determined from triplicate wells. Asterisks indicate that the differences in viral titer between experimental groups are statistically significant when data are compared using one-way analysis of variance (ANOVA) with Tukey’s multiple-comparison procedure (*, *P* < 0.05; **, *P* < 0.01; ***, *P* < 0.001). Dotted lines indicate the limit of detection (100 TCID_50_/ml). Statistical analyses were performed as follows. (i) For the 33°C panel, the virus titers for Cal/09 WT were statistically significantly different from all other viruses at 24 h (*P* < 0.05). The virus titers for Cal/09 AA and Cal Len (PR8) were statistically significantly different from each other at 48 h (*P* < 0.05). (ii) For the 37°C panel, the virus titers for Cal/09 WT were statistically significantly different from all other viruses at 12 and 24 h (*P* < 0.05). The virus titers for Cal/09 Len and Cal Len (PR8) were statistically significantly from each other at 24 h (*P* < 0.05). The virus titers for Cal/09 WT were statistically significantly different from Cal Len at 48 h (*P* < 0.05). (iii) For the 39°C panel, the virus titers for Cal/09 WT were statistically significantly different from all other viruses at all time points (*P* < 0.05, except for the 12-h time point, where *P* < 0.001).

Influenza A virus (IAV) infects 50 million people every year in the United States, leading to between 2,000 and 49,000 annual deaths (1, 2). The last influenza season alone saw over 700,000 hospitalizations and more than 80,000 deaths (3, 4). Currently, both an inactivated influenza vaccine (IIV) and a live attenuated influenza vaccine (LAIV) are licensed and recommended for the prevention of influenza infection in the United States. (5). Seasonal formulations for both IIVs and LAIVs are developed each year to contain the surface antigens, hemagglutinin (HA) and neuraminidase (NA), of the predicted predominantly circulating strains of IAV (6) (Fig. 1A). Nonetheless, vaccine efficacy for seasonal influenza ranges only between 40 and 60%, which is far less than for most other common vaccines (7, 8).

While humoral immunity is the predominant goal of current vaccination strategies, the T-cell response is also important. The presence of influenza subtype-specific CD8 T cells reduces both the severity and duration of IAV infections in humans, and mouse studies have shown an influenza-specific T-cell response is required for viral clearance in the lungs (9, 10). Furthermore, resident memory CD8 T cells (TRM) have been shown to underlie heterosubtypic immunity (i.e., antibody-independent immunity to a novel influenza virus, in mice previously infected with a different strain of influenza [11–13]). CD8 TRM cells generated by a single IAV infection have limited longevity in the lungs compared to CD8 TRM cells in other tissues, corresponding to a waning in heterosubtypic immunity (12, 14). However, recent studies have demonstrated that repeated antigen exposure extends the durability of lung CD8 TRM cells and that CD8 TRM cells in the upper respiratory tract not only greatly exceed the longevity of lung CD8 TRM cells but also are independently capable of preventing pulmonary influenza infection (15, 16).

One of the theoretical benefits of LAIVs over IIVs is that LAIVs generate IAV-specific T-cell immunity, owing to the fact that LAIV is a live replicating virus (17–23). However, on average over multiple influenza seasons, there is no superiority of LAIV compared to IIV in adults, even in years when vaccine surface proteins for both LAIV and IIV were poor antigenic matches for the circulating strains, and one might have expected to see at least partial protection by T-cell responses to conserved viral internal proteins (24). One possible reason for this unexpected observation is that the vaccine master donor virus (MDV) that provides the six internal gene segments other than HA and NA has been unchanged since LAIV was developed by cold passaging the H2N2 subtype seasonal strain A/Ann Arbor/6/60 H2N2 (AA/60) in 1960 (25) (Fig. 1A). H2N2 subtype IAV has not circulated as a seasonal strain since it was supplanted by H3N2 subtype IAV in 1968 (26). In addition, the internal, i.e., non-HA and non-NA, gene segments of current seasonal H3N2 and H1N1 strains, which contain the major immunodominant viral T-cell epitopes (27), are significantly different than their H2N2 counterparts at the amino acid level. Thus, generating an LAIV with internal gene segments better matched to currently circulating strains of H1N1 and H3N2 IAV might enhance heterosubtypic immunity and make LAIV a superior vaccine, especially in years with a vaccine/circulating strain antigen mismatch.

Both the AA/60 H2N2 LAIV MDV and the LAIV MDV licensed for use in Russia, A/Leningrad/134/17/57 H2N2 (referred to here as “Len”), possess attenuated (*att*) and temperature sensitive (*ts*) phenotypes (28, 29). They replicate well at permissive upper airway temperatures of 33°C due to their cold-adapted (*ca*) phenotype but not at lower airway temperatures of 37°C (28, 29). The amino acid residues conferring these *ts*, *ca*, and *att* phenotypes have been mapped for both viruses and share no residues in common, although they fall almost exclusively into the genes encoding viral RNA-dependent RNA polymerase (28, 29). Moreover, our group and others have introduced the mutations responsible for the *ts* and *att* phenotype of AA/60 H2N2 into different IAV subtype and strain backgrounds and have shown that they confer a *ts* phenotype in cell culture and an *att* phenotype in animal models (30–37).

The 2009 pandemic isolate, A/California/04/2009 (Cal/09), possesses internal gene segments that are almost identical to currently circulating H1N1 strains. Thus, we hypothesized that creating a LAIV in the genetic background of Cal/09 might be an ideal approach to improving this vaccine and eliciting H1N1 subtype-specific T-cell immunity.

We have shown that the *ts* residue signature of AA/60 attenuates Cal/09 by only 10-fold in mice, which is insufficient to give confidence about the safety of such an LAIV (38). However, we have recently demonstrated that an LAIV in the background of the prototypical A/Puerto Rico/8/1934 (PR8) H1N1 virus with the *ts* residue signature of the Russian Len LAIV is >100-fold more *ts* at 37°C and is >4.5-fold more attenuated in mice than a PR8 LAIV with signature *ts* residues from the American Ann Arbor LAIV (39).

In the present study, we therefore introduced the *ts* residue signature of the Russian Len LAIV into the background of Cal/09 (Cal/09 Len). In contrast to Cal/09 AA, Cal/09 Len is both *ts in vitro* and highly *att* in mice, and these phenotypes are unaffected by differences in viral surface proteins assessed. We also show that this novel Cal/09 Len LAIV generates robust humoral immunity at a safe dose and protects mice from a lethal homologous challenge. Finally, we generated a second control LAIV containing identical surface antigens, but internal gene segments that were mismatched to our challenge virus. The novel Cal/09-based LAIV elicited superior (and protective) T-cell-mediated immunity to a heterologous challenge virus, unlike the control LAIV. These finding suggest the feasibility of improving the efficacy of the currently available U.S. (AA) or Russian (Len) LAIVs by better matching the sequence of the MDV to currently circulating viral strains.

## RESULTS

### Generation and phenotypic characterization of Cal/09 Len LAIV.

In order to generate a Cal/09 Len LAIV (Cal/09 Len), we introduced the following four residues previously identified as conferring the *ts* phenotype in the licensed Len MDV into the rescue plasmids of Cal/09: PB2 I478L, PB1 K265N and V591I, and NS2 M100I (Fig. 1B) (29). We next generated two recombinant Cal/09 Len viruses, one with wild-type (WT) Cal/09 HA and NA (Cal/09 Len) and one with the HA and NA of PR8 [Cal/09 Len (PR8)], a much older H1N1 virus strain, containing HA and NA segments that are substantially divergent from the contemporary Cal/09 counterpart. We then performed *in vitro* multicycle viral growth kinetic studies comparing the replication of both Cal/09 Len [Cal/09 Len and Cal/09 Len (PR8)] to WT Cal/09 at 33, 37, and 39°C (Fig. 1C). The previously characterized Cal/09 virus with the mutations responsible for the *ts* phenotype of AA/60 (Cal/09 AA) (38) was also included in the assay.

At 33°C, all LAIV viruses [Cal/09 Len, Cal/09 Len (PR8), and Cal/09 AA) reached viral titers within 1 log (50% tissue culture infective dose [TCID_50_]/ml) of Cal/09 WT at all tested times postinfection (p.i.). At 37°C, Cal/09 WT reached high viral titers similar to those obtained at 33°C, while the three LAIV viruses [Cal/09 Len, Cal/09 Len (PR8), and Cal/09 AA] achieved lower viral titers than at 33°C. Finally, at 39°C, viral replication of Cal/09 WT was similar to that observed at 37°C, while Cal/09 AA viral titers were reduced, as previously described (38). Cal/09 Len (PR8) was only detected at 12 h p.i., and the replication of Cal/09 Len was undetectable at 39°C (Fig. 1C). These results suggest that the amino acid substitutions of the Russian Len MDV LAIV confer a more stringent shutoff temperature to the Cal/09 virus than those of the AA MDV LAIV.

### *In vivo* attenuation of Cal/09 Len LAIV.

**FIG 2:**
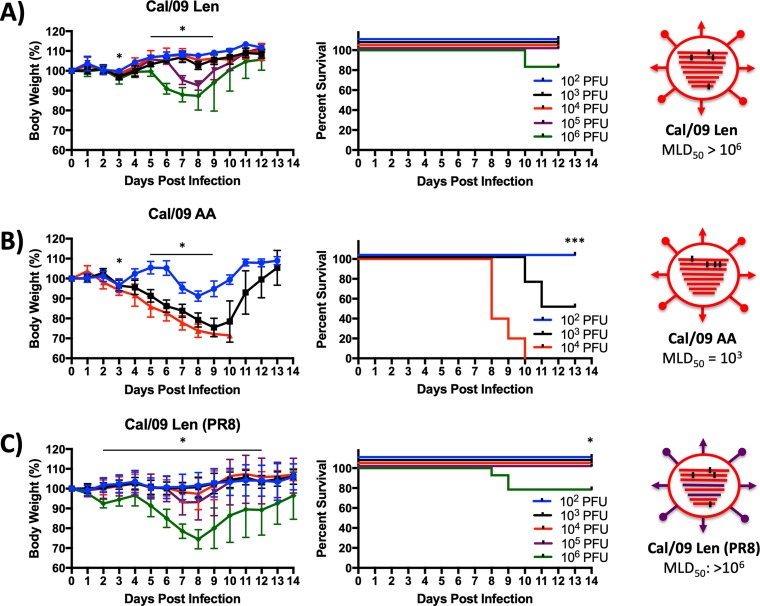
Attenuation of Cal/09Len and Cal/09AA in mice. Mice (see Materials and Methods) were infected i.n. with the indicated PFU of Cal/09 Len (*n* = 4 to 5/group) (A), Cal/09 AA (*n* = 4 to 5/group) (B), and Cal/09 Len (PR8) (*n* = 11/group) (C) recombinant viruses and monitored daily for 2 weeks for body weight (left panels) and survival (right panels). A group size of *n* = 10 to 11 was used for mice infected with Cal/09 Len (PR8) because these same mice were used in the subsequent heterologous challenge experiment (Fig. 5), during which six of the animals were sacrificed before the end of the infection period in order to titer viral replication in lungs and nares. Mice that lost 30% of their initial body weight were sacrificed. Data represent means ± the SD of the results determined for individual mice (*n* = 4 to 11/group). The viral MLD_50_ values were calculated based on the survival data using the method of Reed and Muench. Asterisks indicate that the differences in body weight or survival between experimental groups are statistically significant when data are compared using one-way ANOVA with Tukey’s multiple-comparison procedure (body weight) or log-rank test (survival) (*, *P* < 0.05; ***, *P* < 0.001). Schematic representations of the viruses used to inoculate mice (far right) depict Cal/09 Len (A), Cal/09 AA (B), and Cal/09 Len (PR8) (C). Cal/09 gene segments (red) with *ts* amino acid residues (black) and surface antigens from Cal/09 (red) or PR8 (purple) are shown. Statistical analyses were performed. (A, left) Body weights were statistically significantly different from each other at day 3 (a 10^2^ PFU inoculum compared to a 10^6^ PFU inoculum; *P* < 0.05). Body weights were also statistically significantly from each other at days 5 through 9 (a 10^2^ PFU inoculum compared to a 10^6^ PFU inoculum; *P* < 0.05). (B, left) Body weights in the experimental groups were statistically significantly different from each other at day 3 (a 10^2^ PFU inoculum compared to a 10^6^ PFU inoculum; *P* < 0.05). Body weights were also statistically significantly different from each other at day 5 through 9 (a 10^2^ PFU inoculum compared to a 10^6^ PFU inoculum; *P* < 0.05). (B, right) Survival in the experimental groups was statistically significantly different from each other at day 14 (a 10^2^ PFU inoculum compared to all groups; *P* < 0.001). (C, left) Body weights were statistically significantly different from each other at day 2 through 12 (10^2^ PFU inoculum compared to 10^6^ PFU inoculum; *P* < 0.05). (C, right) Survival in the experimental groups was statistically significantly different from each other at day 14 (10^2^ PFU inoculum compared to 10^6^ PFU inoculum; *P* < 0.05).

We next compared the virulence of our Cal/09 LAIV viruses in mice. Groups of mice were inoculated intranasally with increasing log_10_ doses of the indicated viruses and were monitored for 14 days for weight loss (left panels) and mortality (right panels) (Fig. 2A and B). As expected, based on the difference in *ts* phenotype, Cal/09 Len was more *att* than Cal/09 AA; Cal/09 Len did not induce weight loss below baseline until given at doses of 10^5^ and only began causing mortality at doses of 10^6^ PFU (Fig. 2A), resulting in a theoretical 50% mouse lethal dose (MLD_50_) of >10^6^ PFU. In contrast, Cal/09 AA induced weight loss even at the lowest dose of 10^2^, with a calculated MLD_50_ of ∼10^3^ (Fig. 2B), as we have previously described (38).

**FIG 3:**
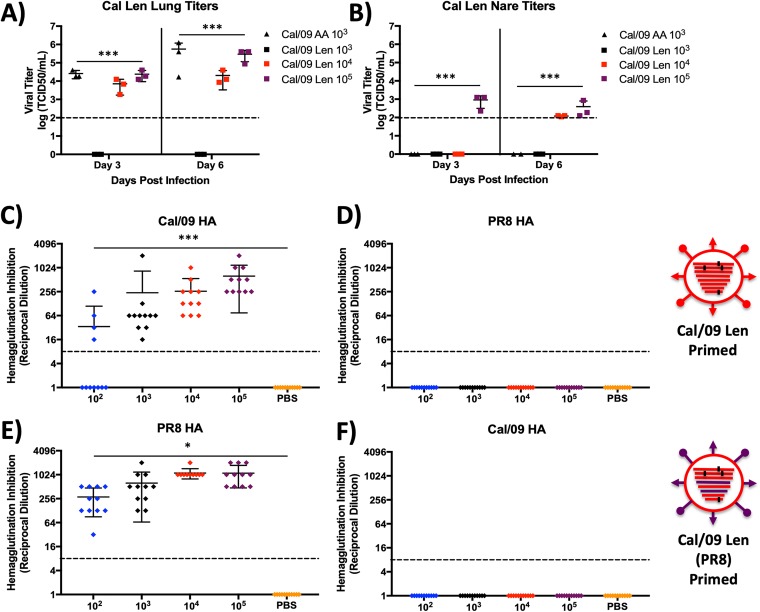
Cal/09 Len replicates at lower levels *in vivo* than Cal/09 AA but still generates a robust antibody response at a safe dose. (A and B) *In vivo* viral replication. Mice (*n* = 3/group) were inoculated i.n. with the indicated doses in PFU of Cal/09 Len or Cal/09 AA. At days 3 and 6 p.i., virus titers (TCID_50_/ml) were determined from total lung (A) and nare (B) homogenates on MDCK cells. The mean virus titers (longest lines) ± the SD (error bars) for each group of mice are indicated. Asterisks indicate that the differences in viral titers between experimental groups are statistically significant when data are compared using one-way ANOVA with Tukey’s multiple-comparison procedure (***, *P* < 0.001). Dotted lines indicate the limit of detection (100 TCID_50_/ml). (C to F) Induction of HAI antibodies. Mice (*n* = 11/group) mice were bled at 14 days postpriming with Cal/09 Len (C and D) or Cal/09 Len (PR8) (E and F). Four hemagglutinating units of WT Cal/09 (C and F) or WT PR8 (D and E) were incubated with 2-fold serial dilutions of serum from Cal/09 Len-vaccinated mice (C and D) or CA/09 Len (PR8)-vaccinated mice (E and F) to determine the ability of the sera to prevent virus from binding to RBCs. A 1/8 dilution is the limit of detection in the assay and is indicated by a dotted line. Each symbol represents the value for an individual mouse, and the mean (longest lines) ± the SD for the groups of mice are also shown. Asterisks indicate that the differences in hemagglutination inhibition between experimental groups are statistically significant when data are compared using one-way ANOVA with Tukey’s multiple-comparison procedure (***, *P* < 0.001). Statistical analyses were performed. (A) Virus titers in lung tissue were statistically significantly different on day 3 and day 6 for the 10^3^ Cal/09 Len inoculum compared to all other groups (*P* < 0.001). (B) Virus titers in the nares were statistically significantly different on day 3 for the 10^5^ Cal/09 Len inoculum compared to all other groups (*P* < 0.001). Virus titers in nares were statistically significantly different on day 6 for the 10^4^ and 10^5^ Cal/09 Len inocula compared to all other groups (*P* < 0.001). (C) HAI titers were statistically significantly different for the 10^3^, 10^4^, and 10^5^ virus inocula compared to the PBS control (*P* < 0.001). (E) HAI titers were statistically significantly different for the 10^2^, 10^3^, 10^4^, and 10^5^ virus inocula compared to the PBS control (*P* < 0.05).

To examine *in vivo* viral replication, we determined the virus titers from the lungs and nares of three mice at both day 3 and day 6 p.i. at several doses of Cal/09 Len (10^3^, 10^4^, and 10^5^ PFU) and one comparable dose of Cal/09 AA (10^3^ PFU) (Fig. 3A and B). In the lungs, Cal/09 Len titers were undetectable at an inoculation dose of 10^3^ PFU, whereas Cal/09 AA at the same dose resulted in high viral lung titers at both day 3 and day 6 p.i. (Fig. 3A). Cal/09 Len titers were detectable at inoculation doses of 10^4^ and 10^5^ PFU, with the 10^5^ dose as high as the Cal/09 AA 10^3^ dose titers on both days 3 and 6 p.i. This was notable given that none of the mice given the 10^5^ dose of Cal/09 Len died or lost weight below baseline, whereas 50% of the mice given the 10^3^ dose of Cal/09 AA died, and the group lost more than 20% of their weight on average (Fig. 2). In the nares, measurable titers of Cal/09 Len were detectable after infection with 10^5^ PFU of virus at both days 3 and 6, but Cal/09 AA titers were undetectable at both time points, despite the use of a virus inoculum approaching the MLD_50_ for this virus (10^3^ PFU) (Fig. 3B).

Taken together, these findings suggest that Cal/09 Len is more attenuated than Cal/09 AA and that the difference in attenuation cannot entirely be explained by differential viral replication in the lungs. Similar results were also observed, although to a lesser extent, in our previous report using the backbone of PR8 (39).

### Cal/09 Len induces robust humoral immunity and is efficacious against homologous challenge a safe dose.

To examine humoral immunity elicited by Cal/09 Len, groups of mice were inoculated with increasing log_10_ doses of virus. Fourteen days later, sera were collected and used to determine hemagglutination inhibition (HAI) titers against WT Cal/09 virus (Fig. 3C). Most mice vaccinated with doses of 10^3^ PFU and all mice inoculated with doses of ≥10^4^ PFU of Cal/09 Len had HAI titers of 1:40 or above (Fig. 3C and D), a threshold generally considered to correspond to a 50% protection from IAV infection (40).

**FIG 4:**
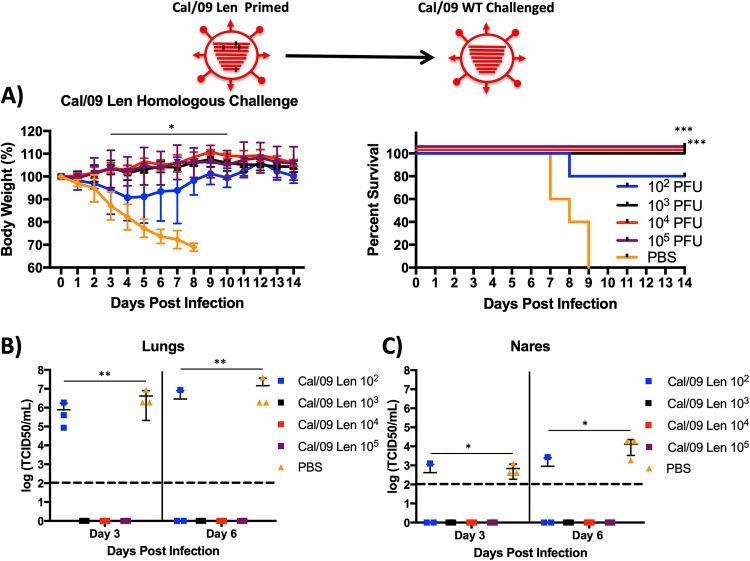
Cal/09 Len protects mice from a lethal homologous challenge at safe doses. Mice (*n* = 11/group) were inoculated i.n. with PBS (orange line) or the indicated doses in PFU of Cal/09 Len (10^2^ to 10^5^). At 3 weeks postpriming, the mice were challenged i.n. with 100 MLD_50_ of a homologous virus (WT Cal/09). For 2 weeks postchallenge, weight loss (A, left panel) (plotted data represent means ± the SD) and survival (A, right panel) were monitored daily (*n* = 5/group). Mice that lost 30% of their initial body weight were sacrificed. Asterisks indicate that the differences in body weight or survival between experimental groups are statistically significant when data are compared using one-way ANOVA with Tukey’s multiple-comparison procedure (body weight) or log-rank test (survival) (*, *P* < 0.05; ***, *P* < 0.001). At days 3 and 6 postchallenge, the challenge virus titer (TCID_50_/ml) was determined from total lung (*n* = 3/group) (B) and nare (*n* = 3) (C) homogenates on MDCK cells. The mean virus titers (longest lines) ± the SD (error bars) for the groups of mice are indicated. Asterisks indicate that the differences in viral titer between experimental groups are statistically significant when data are compared using one-way ANOVA with Tukey’s multiple-comparison procedure (**, *P* < 0.01; ***, *P* < 0.001). Dotted lines indicate the limit of detection (100 TCID_50_/ml). Statistical analyses were performed. (A, left) Body weights in the groups were statistically significantly different from each other at day 3 through 10 (10^3^, 10^4^, and 10^5^ PFU inoculum groups compared to the PBS control; *P* < 0.05). (A, right) Survival in the experimental groups was statistically significantly from each other at day 14 (all inoculum groups, compared to the PBS control; *P* < 0.001). (B) Virus titers in lung tissue were significantly different on day 3 and day 6 for the 10^3^, 10^4^, and 10^5^ Cal/09 Len inocula compared to the PBS control group (*P* < 0.01). (C) Virus titers in nares were significantly different on day 3 and day 6 for the 10^3^, 10^4^, and 10^5^ Cal/09 Len inocula compared to the PBS control group (*P* < 0.5).

We next proceeded with homologous challenge studies using Cal/09 WT. At 21 days after vaccination with Cal/09 Len (and 7 days after bleeding to determine HAI titers), mice were challenged with 100× MLD_50_ of WT Cal/09, and five mice from each group were monitored for 14 days for weight loss (left panel) and mortality (right panel) (Fig. 4A). All mice vaccinated with doses of ≥10^3^ PFU of Cal/09 Len were completely protected from the lethal challenge, as determined by both weight loss and survival (Fig. 4A). Virus titers in lungs and nares of three mice from each group were also measured at days 3 and 6 postchallenge (Fig. 4B and C); mice immunized with ≥10^3^ PFU of Cal/09 Len had undetectable challenge virus titers (Fig. 4B and C). Collectively, these data demonstrate that a well-tolerated safe dose of Cal/09 Len, 10^4^ PFU, induces a robust antibody response capable of completely protecting mice from a lethal homologous viral challenge.

### Designing a challenge virus to test the importance of the internal genes of Cal/09 Len in heterologous protection.

In order to examine heterologous protection induced by Cal/09 Len, we took advantage of the fact that antibodies elicited against PR8 H1N1 HA and NA fail to inactivate the 2009 pH1N1 Cal/09 virus (41). Thus, we used the Cal/09 Len virus with PR8 HA and NA components [Cal/09 Len (PR8)], immunized mice with this virus, and then challenged them with 100× MLD_50_ of WT Cal/09.

As a prelude to these challenge experiments, we first investigated the *in vivo* attenuation of, and humoral immune response to, Cal/09 Len (PR8) in order to ensure it was similarly attenuated to Cal/09 Len and did not induce antibodies reactive to the Cal/09 HA. To this end, groups of mice were inoculated with increasing log_10_ doses of Cal/09 Len (PR8) and then tracked for weight loss and death for 14 days (Fig. 2C). Sera were collected from surviving mice at day 14 p.i. and used to determine HAI titers against the WT PR8 and Cal/09 viruses (Fig. 3E and F).

There was little difference in morbidity or mortality caused by infection with Cal/09 Len (PR8) and Cal/09 Len (Fig. 2A and C). Both viruses did not induce significant weight loss until given at doses of 10^5^ PFU and only began causing mortality at doses of 10^6^ PFU, with a theoretical MLD_50_ of >10^6^ PFU (Fig. 2A and C). Interestingly, at the highest dose level (10^6^ PFU) Cal/09 Len (PR8) caused more pronounced weight loss that Cal/09 Len, even though the viruses were indistinguishable in terms of mortality (Fig. 2A and C). Furthermore, mice inoculated with Cal/09 Len (PR8) developed robust HAI titers against WT PR8 but did not develop measurable HAI titers against WT Cal/09 (Fig. 3E and F), suggesting that there would be no PR8-Cal/09 antibody cross-protection, as was expected based on prior findings (41).

### Heterologous protection elicited by Cal/09 Len is enhanced when the internal genes of the vaccine match the challenge virus.

In order to evaluate the importance of internal genes to heterologous protection elicited by Cal/09 Len, we designed heterologous challenge experiments using Cal/09 WT. As a first step, we compared the protective efficacy of Cal/09 Len (PR8) to a PR8-based LAIV with Len *ts* mutations (PR8/Len) (39). These two H1N1 LAIVs contain identical surface antigens and *ts*-conferring amino acids. However, the internal proteins of PR8 and Cal/09 are significantly divergent, and their differences include immunodominant CD8 T-cell epitopes recognized in C57BL/6 mice (42, 43).

**FIG 5:**
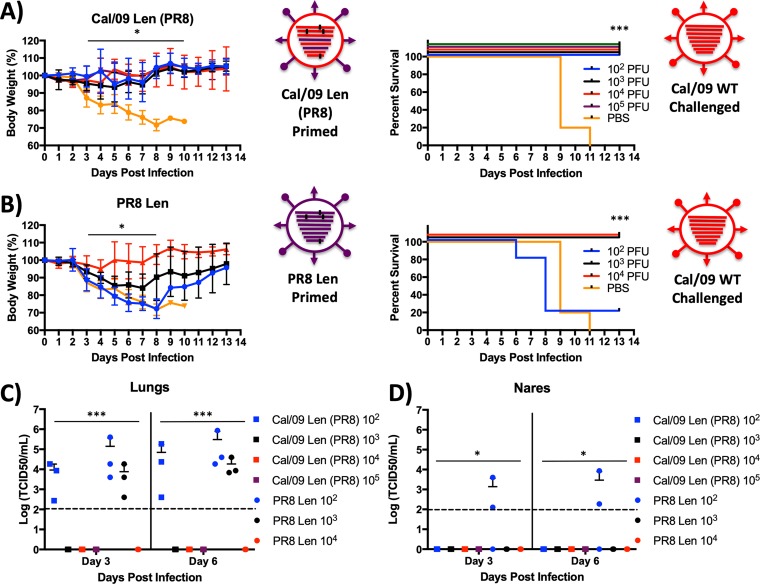
Cal/09 Len confers better protection against a lethal heterologous challenge than PR8/Len. Mice (*n* = 11/group) were inoculated i.n. with PBS (orange line) or with the indicated doses in PFU of Cal/09 Len (PR8) (A) and PR8/Len (B). At 3 weeks postpriming, the mice were challenged i.n. with 100 MLD_50_ of a heterologous virus (WT Cal/09 H1N1). For 2 weeks postchallenge, weight loss (left panels) (plotted data represent means ± the SD) and survival (right panels) were monitored daily (*n* = 5). Mice that lost 30% of their initial body weight were sacrificed. Asterisks indicate that the differences in body weight or survival between experimental groups are statistically significant when data are compared using one-way ANOVA with Tukey’s multiple-comparison procedure (body weight) or log-rank test (survival) (*, *P* < 0.05; ***, *P* < 0.001). At days 3 and 6 postchallenge, the challenge virus titer (TCID_50_/ml) was determined from total lung (*n* = 3) (C) and nare (*n* = 3) (D) homogenates on MDCK cells using the method of Reed and Muench. The mean virus titers (longest lines) ± the SD (error bars) for the groups of mice are indicated. Asterisks indicate that the differences in viral titer between experimental groups are statistically significant when data are compared using one-way ANOVA with Tukey’s multiple-comparison procedure (*, *P* < 0.05; ***, *P* < 0.001). Dotted lines indicate the limit of detection (100 TCID_50_/ml). Statistical analyses were performed. (A, left) Body weights in the groups were statistically significantly different from each other at days 3 through 10 (10^3^, 10^4^, and 10^5^ PFU inoculum groups compared to the PBS control; *P* < 0.05). (A, right) Survival in the experimental groups was statistically significantly from each other at day 14 (all inoculum groups, compared to the PBS control; *P* < 0.001). (B, left) Body weights in the groups were statistically significantly different from each other at days 3 through 8 (10^4^ PFU inoculum group compared to the PBS control; *P* < 0.05). (B, right) Survival in the groups was statistically significantly from each other at day 14 (10^3^ and 10^4^ inoculum groups, compared to the PBS control; *P* < 0.001). (C, left) Virus titers in lung tissue were statistically significantly different on day 3 and day 6 for the 10^3^, 10^4^, and 10^5^ Cal/09 Len (PR8) and the 10^4^ PR8 Len inocula compared to the other groups (*P* < 0.001). (C, right) Virus titers in nares were statistically significantly different on day 3 and day 6 for the 10^2^ PR8 Len inoculum compared to other groups (*P* < 0.05).

We inoculated groups of mice with increasing nonlethal log_10_ doses of either Cal/09 Len (PR8) (Fig. 2C) or PR8/Len (a virus that replicates to comparable titers in lungs of C57BL/6 mice [39]). At 21 days postvaccination, mice were challenged with 100× MLD_50_ of WT Cal/09, and then each group was monitored for 13 days for weight loss (left panels) and mortality (right panels) (Fig. 5A and B). All mice vaccinated with10^2^ PFU Cal/09 Len (PR8) were completely protected from death following challenge with Cal/09 WT (Fig. 5A), whereas 80% of mice vaccinated with 10^2^ PFU of PR8/Len died (Fig. 5B). Furthermore, even at vaccination doses of 10^3^ PFU, mice receiving PR8/Len lost approximately 10% more weight on average than did animals immunized with 10^3^ PFU of Cal/09 Len (PR8) (Fig. 5A and B).

In order to assess the differential effect of the two LAIVs on replication of challenge virus *in vivo*, the lungs and nares of three mice from each group were harvested on days 3 and 6 postchallenge. Challenge virus was detectable in lungs of mice vaccinated with doses of both 10^2^ and 10^3^ PFU of PR8/Len, but only in the 10^2^ PFU group of Cal/09 Len (PR8)-vaccinated mice (Fig. 5C). Similarly, Cal/09 challenge virus was undetectable in nares of Cal/09 Len (PR8)-vaccinated mice but detectable in the 10^2^ PFU group of PR8/Len-vaccinated animals (Fig. 5D) These results demonstrate that a Cal/09-based LAIV (Cal/09 Len) provides enhanced heterosubtypic protection at lower doses against a Cal/09 backbone challenge than did a PR8-based LAIV (PR8 Len) in terms of mortality, weight loss, and *in vivo* replication.

### Enhanced heterologous protection conferred by Cal/09 Len is not antibody mediated.

**FIG 6:**
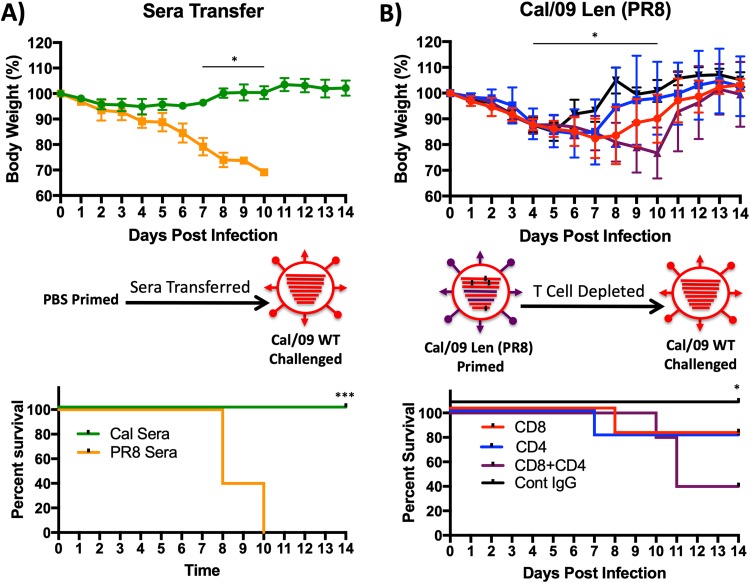
Enhanced heterologous protection conferred by Cal/09 Len is T cell mediated. (A) Cal/09 Len heterologous protection is not antibody mediated. Passive-transfer studies were performed. Mice (*n* = 5/group) were inoculated i.n. with PBS. At 3 weeks postpriming, the mice were challenged i.n. with 10 MLD_50_ of a heterologous virus (WT Cal/09). At 12 h before challenge, PBS mock-vaccinated mice were injected with 400 μl of sera from mice that had been infected with either Cal/09 Len (PR8) (orange) or Cal/09 Len (green). For 2 weeks postchallenge, weight loss (top panel) (plotted data represent means ± the SD) and survival (bottom panel) were monitored daily (*n* = 5/group). Mice that lost 30% of their initial body weight were sacrificed. Asterisks indicate that the differences in body weight or survival between experimental groups are statistically significant when data are compared using one-way ANOVA with Tukey’s multiple-comparison procedure (body weight) or log-rank test (survival) (*, *P* < 0.05; ***, *P* < 0.001). (B) Heterologous protection is decreased in T-cell-depleted mice. Mice (*n* = 5/group) were inoculated i.n. with 10^3^ PFU of Cal/09 Len (PR8). At 3 weeks postpriming, the mice were challenged i.n. with 100 MLD_50_ of a heterologous virus (WT Cal/09). At 6, 4, and 2 days prechallenge and at day 2 postchallenge, groups of mice (*n* = 5) were injected with 200 μg of αCD8 (red) or αCD4 (blue), a combination of 200 μg of both αCD8 and αCD4 (purple), or a nonmouse control IgG antibody (black). For 2 weeks postchallenge, weight loss (top panel) (plotted data represent means ± the SD) and survival (bottom panel) were monitored daily (*n* = 5/group). Mice that lost 30% of their initial body weight were sacrificed. Asterisks indicate that the differences in body weight or survival between experimental groups are statistically significant when data are compared using one-way ANOVA with Tukey’s multiple-comparison procedure (body weight) or log-rank test (survival) (*, *P* < 0.05; ***, *P* < 0.001). Statistical analyses were performed. (A, upper) Body weights in the two groups were statistically significantly different from each other at days 7 through 10 (*P* < 0.05). (A, lower) Survival in the two groups was statistically significantly different from each other at day 14 (*P* < 0.001). (B, upper) Body weights in the groups were statistically significantly different from each other at days 4 through 10 (CD4/CD8-depleted group compared to the control IgG group; *P* < 0.05). (B, lower) Survival in the groups was statistically significantly different at day 14 (CD4/CD8-depleted group compared to the control IgG group; *P* < 0.05).

As previously discussed, antibodies developed against PR8 H1N1 do not protect against Cal/09 H1N1 (41). To ensure that the enhanced heterologous protection observed with Cal/09 Len was not mediated by antibodies, passive-transfer studies were performed. We inoculated groups of mice with phosphate-buffered saline (PBS) and 21 days later, all mice were challenged with 10× MLD_50_ WT Cal/09 (Fig. 6A). Prior to challenge, mice received sera from animals infected with either Cal/09 Len or Cal/09Len (PR8). As expected, sera transferred from Cal/09 Len-infected mice completely protected mice from an otherwise lethal challenge with Cal/09 WT (Fig. 6A). However, sera from Cal/09 Len (PR8)-infected mice had no such protective effect (Fig. 6A), consistent with the fact that anti-PR8 HA antibodies do not bind Cal/09 HA (Fig. 3E and F) and are not protective against WT Cal/09 infection (41). These results affirmed that the heterologous protection achieved by Cal/09 Len was not mediated by antibodies or serum components.

### T-cell responses underlie the enhanced heterologous protection conferred by Cal/09 Len.

**FIG 7:**
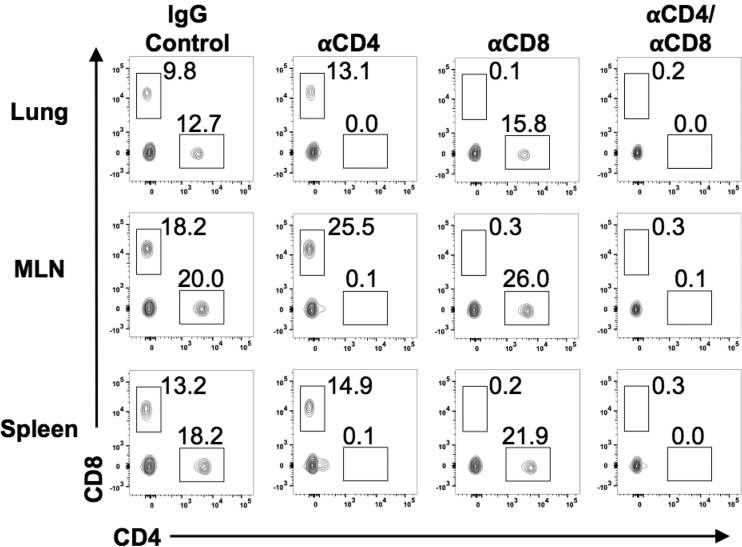
Depleting antibodies effectively diminish T-cell subsets. Cells from lungs (top row), MLNs (middle row), and spleens (bottom row) of mice (*n* = 1 to 2) were examined by flow cytometry for the presence of CD8 and CD4 T cells 1 day after WT Cal/09 challenge (following Cal/09 Len vaccination and the indicated antibody treatment). Representative flow cytometry plots show the percentages of viable lymphoid cells.

To determine whether the enhanced heterologous protection observed with Cal/09 Len was mediated by T cells, we performed an additional heterologous challenge experiment in mice depleted of CD4, CD8, or both CD4 and CD8 T cells (Fig. 6B). We inoculated groups of mice with 10^3^ PFU of Cal/09 Len (PR8); 21 days later, the mice were challenged with 100× MLD_50_ WT Cal/09. Prior to challenge, mice received anti-CD8, anti-CD4, both anti-CD8 and anti-CD4, or control anti-rat IgG monoclonal antibodies. T-cell depletion was verified by flow cytometry (Fig. 7).

Mice depleted of either CD8 or CD4 T cells lost more weight after WT Cal/09 challenge than vaccinated mice treated with the control anti-rat IgG antibody, and one mouse from the CD8- and CD4-depleted group died (Fig. 6B). However, mice depleted of both CD8 and CD4 T cells lost the greatest amount of weight of any vaccinated group and experienced the highest level of mortality (Fig. 6B). Taken together, with the results of our serum transfer experiment, these data suggest that the superior heterologous protection to pH1N1 Cal/09 virus conferred by vaccination with Cal/09 Len (PR8) compared to PR8/Len is due to T-cell immunity.

## DISCUSSION

T-cell-mediated immunity is critical for protective immune responses against natural IAV infection (9–16), but current influenza vaccines elicit poor CD8^+^ T-cell responses (44). Despite this, few studies have attempted to enhance T-cell immunity in an IAV vaccine, and those that have have focused principally on a single protein component of IAV (45–48). Much greater efforts have been directed toward the generation of broadly reactive (and broadly neutralizing) antibody responses (49, 50).

In this work, we addressed the question of whether the effectiveness of LAIV can be enhanced by using an MDV with internal components that better match a potential challenge virus. In order to do this, it was necessary to select a set of *ts* mutations capable of conferring a live-attenuated phenotype on the desired MDV backbone. Previous studies have shown that the *ts* mutations from AA/60 attenuate PR8 significantly in mice but that they fail to attenuate Cal/09 in mice and also fail to attenuate various strains of avian influenza viruses (AIVs) in birds (30, 38, 51). This lack of attenuation in the genetic background of Cal/09 and AIV can be overcome by the addition of other *ts* or *att* mutations (e.g., PB1 319Q [52]) or by the insertion of an attenuating PB1 HA epitope tag (53, 54). However, it is clear that the AA/60 *ts* mutations alone do not reproducibly confer a *ts* or *att* phenotype across diverse viral genetic backgrounds. In contrast, previous reports suggest that the *ts* mutations from Len LAIV may confer a more robust *ts* or *att* phenotype across a variety of viral backgrounds (29, 39). As a result, we chose to use the Len LAIV mutations to attenuate our MDV in the present study.

The studies described here were intended to address this knowledge gap and to examine the phenotypic characteristics and protective efficacy of an LAIV that was created in the backbone of a contemporary H1N1 IAV, Cal/09.

We show that the *ts* mutations from the Russian Len LAIV confer a much more robust *ts* phenotype and a much greater degree of *in vivo* attenuation in mice (by 3 log_10_) to Cal/09 than do the mutations from the U.S. AA/60 LAIV (Fig. 1C and 2) (38). Nevertheless, a Cal/09 virus containing the Len *ts* mutations (Cal/09 Len) retains the ability to replicate in the lungs and nares of mice and elicits a robust antibody response, including HAI antibodies (Fig. 3A to C), that results in protection against a lethal homologous challenge (Fig. 4). Most importantly, Cal/09 Len provides superior T-cell-mediated immunity to a heterologous challenge virus, as measured by morbidity, mortality, and a reduction in challenge virus replication, compared to a control LAIV containing internal components mismatched to the challenge virus (Fig. 5); moreover, this protection is T cell mediated (Fig. 6).

It is noteworthy that the currently licensed U.S. LAIV contains the internal gene segments of a H2N2 MDV that circulated almost 60 years ago (AA/60). The use of this H2N2 MDV backbone is unlikely to be optimal in terms of eliciting strong and broadly reactive T-cell responses against currently circulating H1N1 or H3N2 IAV strains. Rather, our findings suggest that a better approach may be to reengineer the MDV for future LAIVs, such that it contains the internal segments of a more contemporary virus (e.g., Cal/09).

Currently, two distinct lineages of LAIV MDV are licensed for human use: the U.S. AA MDV and the Russian Len MDV. Only the *ts* and *att* mutations of the Russian Len MDV were able to confer a strong *in vitro ts* phenotype and a strong *in vivo att* phenotype to Cal/09. It is still unclear why the mutations responsible for the *ts* and *att* phenotypes of the U.S. AA MDV failed to strongly attenuate Cal/09.

While we have demonstrated that the Len *ts* mutations make Cal/09 highly attenuated in mice, further studies will be required to determine whether a similar level of attenuation is observed in other animal models (e.g., ferrets), while still permitting virus replication at levels capable of inducing robust humoral and cellular immunity. Future experiments in ferrets would also accomplish two additional goals. First, they would test our hypothesis in an animal model that is more genetically outbred than C57/BL6 mice, which is important in terms of the diversity of the influenza-specific CD8^+^ T-cell response (55, 56). Second, such studies would allow us determine whether our findings can be reproduced in the background of H3N2 subtype viruses, which do not replicate in mice.

Finally, before our findings could be translated into human clinical studies, it will of course be necessary to test the safety and immunogenicity of Cal/09 Len in humans. However, even if Cal/09 Len should unexpectedly fail to demonstrate acceptable levels of attenuation in humans, there are multiple other methods that can be applied to rationally attenuate a more contemporary IAV and to produce an LAIV that elicits a more effective cellular immune response (57–60). Likewise, a similar approach can be used for the development and implementation of a new MDV LAIV for the treatment of IAV in other animals.

In summary, these studies show that the *ts* mutations of the Russian Len MDV LAIV confer a strong *ts* and *att* phenotype on a currently circulating H1N1 IAV strain (Cal/09). They further demonstrate that a Cal/09-based LAIV MDV based on the Len mutations induces superior heterologous protection to a WT Cal/09 challenge compared to a control LAIV containing mismatched internal genes. These findings may have important implications for the development of improved LAIVs for the prevention of influenza viral infections in humans, including the development of universal vaccines, as well as other animal species.

## MATERIALS AND METHODS

### Cells and viruses.

Human embryonic kidney 293T (HEK293T cells, ATCC CRL-3216) and Madin-Darby canine kidney (MDCK; ATCC CCL-34) cells were grown in Dulbecco modified Eagle medium (DMEM) (Mediatech, Inc.) supplemented with 10% fetal bovine serum (FBS; Atlanta Biologicals) and 1% penicillin (100 U/ml)–streptomycin (100 μg/ml)–2 mM l-glutamine (P-S-G; Mediatech, Inc.) at 37°C in air enriched with 5% CO_2_. After viral infections, cells were maintained at 37°C in p.i. media: DMEM supplemented with 0.3% bovine serum albumin (Sigma), 1% P-S-G, and TPCK (tolylsulfonyl phenylalanyl chloromethyl ketone)-treated trypsin (2 μg/ml; Sigma).

Recombinant A/California/4_NYICEE3/2009 H1N1 (Cal/09) wild-type (WT) and Cal/09 containing the temperature-sensitive (*ts*) mutations of the A/Ann Arbor/6/60 H2N2 MDV LAIV (Cal/09 AA) have been previously described (38). The recombinant Cal/09 viruses with the *ts*, *ca*, and *att* mutations from A/Leningrad/134/17/57 and the HA and NA from Cal/09 (Cal/09 Len) or PR8 [Cal/09 Len (PR8)] were generated using plasmid-based reverse genetics techniques at 33°C (61).

The recombinant A/Puerto Rico/8/34 H1N1 (PR8) virus containing the *ts*, *ca*, and *att* mutations of the Russian MDV A/Leningrad/134/17/57 MDV (PR8/Len) was generated using previously described plasmid-based reverse techniques (61) and is described elsewhere (39). All viral rescues, stocks, and titrations were conducted at 33°C (LAIV) or 37°C (WT).

### Plasmids.

Cal/09 Len PB1, PB2, and NS plasmids were generated by site-directed mutagenesis (Agilent) using the Cal/09 ambisense pDZ WT plasmids with the following mutations: PB1 I487L, PB2 K265N and V591I, and NS2 M100I. All plasmids were confirmed by sequencing (ACGT, Inc.). Primers for the generation of the different plasmid constructs are available upon request.

### Rescue of recombinant Cal/09 viruses.

Recombinant viruses were rescued as previously described, and propagated in cell lines (61). Cocultures (1:1) of HEK293T and MDCK cells in six-well plates were transiently cotransfected in suspension with 1 μg of each of the ambisense plasmids (pDZ-PB2_Cal/09 Len_, PB1_Cal/09 Len_, PA_Cal/09 WT_, HA_Cal/09 WT_ or HA_PR8 WT_, NP_Cal/09 WT_, NA_Cal/09 WT_ or NA_PR8 WT_, M_Cal/09 WT_, and NS_Cal/09 Len_) using Lipofectamine 2000 (Invitrogen). At 12 h posttransfection, the transfection medium was replaced with p.i. medium. At 72 h posttransfection, tissue culture supernatants (TCS) were collected, clarified, and used to infect fresh MDCK cells (1 × 10^6^ cells/well, six-well plate format). At 3 to 4 days p.i., recombinant viruses were plaque purified and propagated in MDCK cells (59). Stocks were titrated by plaque assay (PFU/ml) on MDCK cells as previously described (38). Virus stocks were confirmed by sequencing (ACGT, Inc.) the PB2, PB1, and NS viral segments using purified total RNA (TRIzol reagent; Invitrogen) from infected MDCK cells (1 × 10^6^ cells/well, six-well plate format).

### Virus growth kinetics.

To determine virus growth kinetics *in vitro*, triplicate wells of confluent monolayers of MDCK cells (1 × 10^6^ cells/well, six-well plate format) were infected at a multiplicity of infection (MOI) of 0.001. After 1 h at room temperature, the cells were overlaid with p.i. medium and incubated at 33, 37, or 39°C. At the indicated times (12, 24, 48, and 72 h p.i.), TCS were collected, and viral titers were determined by tissue culture infectious dose assay (TCID_50_/ml) as described previously (52). Mean values and standard deviations (SD) were calculated using Prism software.

### Mouse experiments.

All mouse experiments were conducted using female 6- to 8-week-old C57BL/6 mice purchased from Jackson Laboratories and maintained in the animal care facility at the University of Rochester under specific-pathogen-free conditions. All animal protocols were approved by the University of Rochester Committee of Animal Resources and complied with the recommendations in the *Guide for the Care and Use of Laboratory Animals* of the National Research Council (62). To evaluate the *in vivo* attenuation of the different viruses, mice were anesthetized intraperitoneally (i.p.) with 2,2,2-tribromoethanol (Avertin; 240 mg/kg [body weight]) and then inoculated intranasally (i.n.) with 30 μl of a virus preparation containing the indicated doses of virus diluted in PBS. Mice were monitored daily for 13 to 14 days for morbidity (body weight loss) and mortality (survival). Mice showing a 30% loss in initial body weight were considered to have reached the experimental endpoint and were humanely euthanized. The 50% mouse lethal dose (MLD_50_) was determined using the method of Reed and Muench (63).

For prime-challenge experiments, mice were rested for 21 days between prime and challenge infections, during which time they were also monitored for weight loss and survival. Virus replication was evaluated by determining viral titers in the lungs and nares of infected mice at 3 and 6 days p.i. To that end, three mice in each group were sacrificed, and the lungs and nares were extracted and homogenized. Virus titers were determined by TCID_50_ as indicated above.

### Passive serum transfer.

Individual serum pools were obtained from mice 3 weeks after infection with 10^5^ PFU of the indicated viruses. Each serum pool was then injected i.p. in a volume of 400 μl into naive mice. At 12 h after injection, mice were anesthetized (i.p.) and challenged (i.n.) with 30 μl of the indicated challenge virus. The mice were monitored daily for weight loss and survival as described above.

### T-cell depletion.

For T-cell depletion, mice were primed with Cal/09 Len (PR8) and rested for 21 days. Groups of mice were then injected i.p. with 200 μg of an anti-CD4 antibody (clone GK1.5; BioXCell), 200 μg of an anti-CD8 antibody (clone 2.43; BioXCell), 200 μg of both anti-CD4 and anti-CD8 antibodies, or 200 μg of an IgG2b isotype antibody control (clone LTF-2; BioXCell). Antibodies were injected every other day three times before viral infection and once at day 2 after infection with Cal/09 WT. After infection, the mice were monitored daily for weight loss and survival as indicated above.

### HAI assays.

Mouse sera were treated with receptor-destroying enzyme (RDE; Denka Seiken) and heat inactivated for 30 min at 56°C. Sera were then serially 2-fold diluted (starting dilution, 1:8) in 96-well V-bottom plates and mixed 1:1 with four hemagglutinating units of either Cal/09 WT or PR8 WT virus for 60 min at room temperature. HAI titers were determined by adding 0.5% turkey red blood cells (RBCs) to the virus-antibody mixtures for 30 min on ice, as previously described (59).

### Flow cytometry.

On day 8 after infection with Cal/09 WT, lungs, mesenteric lymph nodes (MLNs), and spleens were collected and processed to obtain cellular preparations. Lungs were perfused with PBS, surgically removed, and dissociated in C tubes by the GentleMACS (Miltenyi Biotec) using the Lung01 program. Samples were then incubated in 5 ml (2 μg/ml) of collagenase II in RPMI medium containing 8% FBS for 30 min at 37°C with gentle shaking. After digestion, the samples were further dissociated using the Heart01 program. Cell suspensions were filtered through 100-μm filters prior to 75:40 Percoll (GE Healthcare) discontinuous gradient separation. The top layer, containing fat and other debris, was removed by aspiration. The cell layer was harvested and washed prior to counting and staining. Single-cell suspensions were prepared from collected spleens and MLNs by disruption in RPMI plus 8% FBS. Counting was achieved through trypan blue exclusion on a hemocytometer.

To prepare samples for flow cytometry analysis, single-cell suspensions in PBS were stained with purified CD16/32 (clone 2.4G2), the fixable viability dye Live Dead Aqua (Invitrogen), and the following antibodies: TCRβ-Brilliant Violet 786 (clone H57-597), CD8α-PE (clone 53-6.7), and CD4-APC (clone RM4-5). All antibodies were obtained from BD Biosciences or BioLegend. Cells were analyzed by an LSRII (BD Biosciences) in the University of Rochester Flow Cytometry core facility and analyzed by using FlowJo software (Tree Star).
